# CAR-NK cell therapy for glioblastoma: what to do next?

**DOI:** 10.3389/fonc.2023.1192128

**Published:** 2023-06-19

**Authors:** Qi Xiong, Jiao Zhu, Yong Zhang, Hongxin Deng

**Affiliations:** Department of Biotherapy, Cancer Center and State Key Laboratory of Biotherapy, West China Hospital, Sichuan University, Chengdu, China

**Keywords:** CAR-NK cells, glioblastoma, natural killer cells, immunotherapy, cell therapy

## Abstract

Glioblastoma is a malignant tumor with the highest morbidity and mortality in the central nervous system. Conventional surgical resection combined with radiotherapy or chemotherapy has a high recurrence rate and poor prognosis. The 5-year survival rate of patients is less than 10%. In tumor immunotherapy, CAR-T cell therapy represented by chimeric antigen receptor-modified T cells has achieved great success in hematological tumors. However, the application of CAR-T cells in solid tumors such as glioblastoma still faces many challenges. CAR-NK cells are another potential adoptive cell therapy strategy after CAR-T cells. Compared with CAR-T cell therapy, CAR-NK cells have similar anti-tumor effects. CAR-NK cells can also avoid some deficiencies in CAR-T cell therapy, a research hotspot in tumor immunity. This article summarizes the preclinical research status of CAR-NK cells in glioblastoma and the problems and challenges faced by CAR-NK in glioblastoma.

## 1 Introduction

Glioblastoma is a malignant tumor with the highest morbidity and mortality in the central nervous system. The incidence of malignant brain tumors is 29.7% in all brain and other central nervous system tumors. Among them, the incidence of glioblastoma is 14.5%, accounting for 48.6% of the incidence of malignant brain tumors, which is the highest incidence of malignant brain tumors (1). According to the WHO tumor grading standard, glioblastoma is the most malignant CNS WHO grade 4 malignant tumor (2). Epidemiological statistics show that the median survival time of patients with glioblastoma is 8 months, and the 5-year survival rate is less than 10%. It is the most lethal malignant brain tumor (1) (3). The traditional treatment of glioblastoma is surgical resection, followed by radiotherapy and chemotherapy. This standard treatment can partially alleviate the disease progression of patients and improve the quality of life of patients. However, over 90% of patients will have tumor recurrence after standard treatment (4) (5). With the breakthrough of immunotherapy technology represented by PD-1 antibody and CAR-T cells, new hope has been brought to treat malignant tumors (6). Various immunotherapy strategies have also been developed to treat glioblastoma (7) (8). CAR-T, namely chimeric antigen receptor T cell. The activated T cells *in vitro* are modified by the chimeric antigen receptor gene to form CAR-T cells. CAR-T cells target tumor-associated antigens through their expressed chimeric antigen receptors, specifically recognizing and killing tumor cells (9) (10). Due to the excellent anti-tumor effect of CAR-T cells, the United States first approved two CAR-T drugs for treating non-Hodgkin’s lymphoma and acute lymphoblastic leukemia in 2017. By March 2023, eight CAR-T drugs on the market worldwide, all for treating hematological malignancies. In terms of clinical trials, as of March 2023, 19 clinical trials of CAR-T in the treatment of glioblastoma were carried out worldwide (https://clinicaltrials.gov/). Although CAR-T has achieved great success in hematological tumors, it still faces many challenges in treating solid tumors such as glioblastoma (11) (12). Studies have shown that T cells in the tumor immune microenvironment of glioblastoma are mainly Treg cells and exhausted cytotoxic T cells (13). Therefore, CAR-T has a natural immunosuppressive effect in the treatment of glioblastoma. Combining CAR-T and immune checkpoint inhibitors such as PD-1 antibodies may be an effective solution strategy (9). However, CAR-T cell therapy may cause side effects such as anti-host immune disease, neurotoxicity and cytokine storm. In addition, CAR-T cell therapy also faces many challenges, such as tumor heterogeneity, off-target effect and low tumor infiltration efficiency (14) (15) (16).

NK cells are an essential part of cellular immunity and have antiviral and anti-tumor effects. NK cell killing target cells depends on the dynamic balance of activating and inhibitory receptors on their cell membranes, without needing antigen pre-sensitization and MHC molecule restrictions (17). Compared with T cells, NK cells have the killing function of tumor cells and play an important role in the shaping of innate immunity and acquired immunity (18) (19). NK cells have the characteristics of universal allogeneic source effector cells. CAR-NK cell uses NK cells as effector cells to express chimeric antigen receptors. After optimization of the chimeric antigen receptor domain, CAR-NK cell can exhibit anti-tumor effects similar to CAR-T cells (20) (21). Clinical studies have reported that the response rate of patients treated with CAR-NK cells reached 73%, and no adverse effects, such as cytokine storm, neurotoxicity and anti-host immune disease occurred during the treatment (22). Compared with CAR-T cell, CAR-NK cell has better safety. As of March 2023, only one clinical trial of CAR-NK cells in the treatment of glioblastoma (NCT03383978). CAR-NK cell is another potential immunotherapy strategy for glioblastoma after CAR-T cell. Based on this, this article reviews the preclinical research progress of CAR-NK in glioblastoma and summarizes the problems and challenges faced by CAR-NK in glioblastoma.

## 2 Preclinical study of CAR-NK cells in the treatment of glioblastoma

In the preclinical study of CAR-NK cells in glioblastoma, NK cells have multiple sources (**Table 1**). Tumor-derived cell line NK92 cells are more widely used. In addition, some studies used peripheral blood-derived NK cells as effector cells. The clinical trial of CAR-NK cell in treating glioblastoma (NCT03383978) used NK92 cells as effector cells. Compared with peripheral blood-derived NK cells, NK92 cells are an immortalized cell line derived from tumor patients. The expansion of NK92 cells *in vitro* does not require special culture conditions and is convenient for large-scale preparation (33) (34). In addition, NK92 cells lack the expression of inhibitory receptors KIRs and have low immunogenicity (35). Although tumor-derived NK92 cells have the risk of tumorigenicity, γ-ray irradiation or low-energy electron irradiation can effectively inhibit the proliferation of NK92 cells and maintain their cell activity in the short term. Therefore, CAR-NK cells constructed using NK92 as effector cells have the feasibility of clinical transformation (36) (37) (38). Studies have shown that in the xenograft tumor model of the brain *in situ* glioblastoma in immunodeficient mice, CAR-NK cells constructed with NK92 as effector cells can effectively alleviate the tumor burden of model mice and prolong the survival time of mice (23) (24) (25). Compared with NK92 cells, treating peripheral blood-derived NK cells does not require irradiation of cells, and the risk of tumorigenesis is lower (35). Treatment with chimeric antigen receptors modified by peripheral blood-derived NK cells can also effectively alleviate the tumor burden and prolong the survival of model mice (30) (32). The above studies have shown that NK92 cells and peripheral blood-derived NK cells have good application prospects in glioblastoma. Nevertheless, studies have shown that CAR-NK92 cells have more potent anti-tumor cytotoxicity than CAR-PBNK cells, and CAR-NK92 cells have stronger side effects on non-tumor cells (39). In addition, studies have also found that CAR-NK92 cells secrete higher granzyme A and IL-17A, while CAR-PBNK cells secrete more TNFα, IFNγ and Granulysin cytokines (39). These studies have shown that NK cells from different sources have different characteristics, and it is necessary to select reasonable effector cells in treating glioblastoma with CAR-NK.

**Table 1:** Preclinical studies with CAR-NK cells in glioblastoma.

Targets	Sources	Hinge	Transmembrane domain	Costimulatory domain	Ref.
Her2	NK92	CD8α	CD28	CD28-CD3ζ	(23)
EGFR EGFRvIII	NK92	CD8α	CD28	CD28-CD3ζ	(24)
EGFR EGFRvIII	NK92 NKL	CD8α	CD28	CD28-CD3ζ	(25)
EGFRvIII	YTS	c-Myc-tag	DAP12	DAP12	(26)
EGFRvIII	KHYG-1	CD8α	CD28	CD28-4-1BB-CD3ζ	(27)
EGFRvIII	KHYG-1	CD8α	CD28	CD28-4-1BB-CD3ζ	(28)
B7-H3	NK92	CD8α	CD28	CD28-CD3ζ	(29)
EGFR	PB-NK	CD8α	CD28	CD28-CD3ζ	(30)
c-Met AXL	KHYG-1	CD28	CD28	CD28-CD3ζ	(31)
CD73 GD2 NKG2DL	NK92 PBNK	CD8α	CD28	CD28-CD3ζ	(32)

In addition, there are also significant differences in chimeric antigen receptors used to modify NK cells, which have been reported from the first generation to the third generation (**Table 1**). With the development of CAR-T cells, CAR-T cell technology has developed to the fifth generation, of which the second generation CAR-T is the most classic, and the second generation CAR-T is also the most commonly used type in CAR-T clinical trials (40). The second generation of CAR-T cells with CD28 or 4-1BB combined with CD3ζ into chimeric antigen receptor T cells containing two costimulatory domains (14). Among them, CAR-T cells with CD28-CD3ζ costimulatory domain showed faster and more robust signal activity, while CAR-T cells with 4-1BB-CD3ζ costimulatory domain expressed more genes related to T cell memory (41). In terms of anti-tumor effect, CAR-T cells with 4-1BB-CD3ζ costimulatory domain have a stronger anti-tumor effect (42) (43). However, in the study of CAR-NK cells treatment of glioblastoma, chimeric antigen receptors containing CD28-CD3ζ costimulatory domain are mainly used, and some chimeric antigen receptors containing CD28-4-1BB-CD3ζ costimulatory domain are used (**Table 1**). CAR-NK cells with these two structures can effectively inhibit the progression of glioblastoma in mice (23) (24) (27). The difference between CAR-NK cells with these two structures in anti-glioblastoma is unclear. Studies have shown that CAR-T cells using the CD28-CD3ζ costimulatory domain have a more potent anti-tumor effect than CAR-T cells using the CD28-4-1BB-CD3ζ costimulatory domain (44). Mechanistically, compared to the third-generation CAR-T cells, the second-generation CAR-T cells can activate additional CD3ζ signals, thereby enhancing TCRs signals (45). However, in the ovarian cancer xenograft model, CAR-NK cells with CD28-4-1BB-CD3ζ costimulatory domain have a more potent anti-tumor effect than CAR-NK cells with CD28-CD3ζ costimulatory domain (46). This conclusion is contrary to the above findings in CAR-T. This also reflects the different anti-tumor effects of NK cells and T cells. We may suggest that in the follow-up study of CAR-NK cells, the corresponding chimeric antigen receptors should be rationally designed according to the characteristics of NK cells to optimize the anti-tumor effect of CAR-NK cells, which also provides ideas for the application of CAR-NK in glioblastoma.

## 3 Targets of CAR-NK cells in the treatment of glioblastoma

Reasonable therapeutic targets are of great significance for the tumor specificity of CAR-NK cell therapy. In the preclinical study of CAR-NK cells in treating glioblastoma, the main therapeutic targets were Her2 and EGFRvIII (**Table 1**). The primary therapeutic targets in the clinical trials of CAR-T in the treatment of glioblastoma are Her2, EGFRvIII and IL-13Rα (11). In addition, some new glioblastoma therapeutic targets, such as CSPG4, also have good application prospects.

### 3.1 Her2

Her2 belongs to the human epidermal growth factor receptor family and is a tyrosine kinase receptor on the cell membrane. Her2 does not contain a ligand recognition domain, which activates intracellular downstream signaling pathways by binding to other EGFR family members to form heterodimers. The activation of Her2 affects cell proliferation, differentiation and adhesion (47) (48). High expression of Her2 has been found in various solid tumors such as breast cancer, ovarian cancer and bladder cancer. In addition, Her2 expression is closely related to worse prognosis (49) (50) (51) (52). In early studies, Her2 expression was detected in about 80% of glioblastoma samples (53). However, Her2 expression was not detected in a sample containing 40 cases of glioblastoma (54). In another study, high expression of Her2 was detected in about 40% of 56 glioblastoma samples (23). In a sample study of 107 brain tumors, about 40% of high-grade astrocytomas highly express Her2 (55). The above studies have shown that the expression of Her2 in glioblastoma differs in different patients, which is also closely related to the heterogeneity of tumors (56). Compared with normal tissues, Her2 expression is higher in tumor tissues (57) (58). And Her2 expression is lower in most normal tissues (59).

In 2010, Nabil Ahmed et al. constructed CAR-T cells targeting Her2, which can effectively kill Her2-positive tumor cells derived from glioblastoma patients *in vitro*, effectively inhibit tumor progression in immunodeficient mice and significantly prolong the survival of mice (56). In the subsequent clinical study, after intravenous administration of the CAR-T cells, 7 of the 17 patients enrolled were relieved of tumor progression, and the median survival after treatment was 11.1 months. Although there was no obvious dose-dependent toxicity in this clinical trial, patients after treatment had side effects of headache and spasms (60). This study also suggests that CAR-T cells should further optimize their efficacy and control their negative response in treating solid tumors such as glioblastoma. In a preclinical study of CAR-NK cells, Congcong Zhang et al. analyzed the expression level of Her2 in glioblastoma samples, and high expression of Her2 was detected in about 40% of glioblastomas (23). They used NK92 cells as effector cells to construct Her2-targeted CAR-NK cells, which can effectively kill Her2-positive glioblastoma cell lines and patient-derived primary glioblastoma cells *in vitro*. In the immunodeficiency mouse model, CAR-NK cells can effectively inhibit tumor growth after treatment. The median survival time of CAR-NK treated mice was 200.5 days, about 3 times longer than that of the control group (23). This study shows that CAR-NK cells targeting Her2 are feasible in treating glioblastoma, and related clinical studies are currently underway (NCT03383978).

### 3.2 EGFRvIII

EGFR is expressed on the cell membrane surface and is an epidermal growth factor receptor family member. EGFR recognizes and binds to epidermal growth factor, which induces the receptor to form a homodimer or heterodimer. Then the critical tyrosine residues of the intracellular domain of EGFR to be self-phosphorylated, which activates the downstream signal of EGFR and induces cell proliferation and survival (61). Studies have shown that EGFR expression is higher in glioblastoma and lower in normal brain tissue (62) (63). EGFRvIII is the most common EGFR mutant in glioblastoma, and EGFRvIII mutations can be detected in about 20%-40% of malignant gliomas (64) (65). Structurally, the EGFRvIII mutant lacks EGFR exons 2-7, amino acids 6-273, and glycine (66). Functionally, EGFRvIII lacks an extracellular domain. In the absence of ligands, EGFRvIII can constitutively self-activate, thereby activating tumor-promoting signals and enhancing the tumorigenicity of tumor cells (67) (68) (69). Although the expression of EGFRvIII was not significantly correlated with the survival prognosis of glioblastoma patients, patients with high EGFRvIII expression and increased EGFR expression had a poor survival prognosis (70). In addition, EGFRvIII is a mutant specifically expressed in tumor cells, so EGFRvIII is a tumor-specific therapeutic target.

In 2017, Rouke et al. reported the first clinical trial of CAR-T cells targeting EGFRvIII in treating glioblastoma (NCT02209376) (71). A total of 10 patients with recurrent glioblastoma were enrolled in this clinical trial and treated with a single intravenous infusion of CAR-T cells. After treatment, the tumors of 7 patients were analyzed by surgery. The infiltration of CAR-T cells could be detected in all 7 patients, and the expression of EGFRvIII in 5 patients was decreased. However, after CAR-T cell treatment, the tumor microenvironment increased the expression of inhibitory molecules and the infiltration of Treg cells. This clinical trial shows that although intravenous infusion of CAR-T cells can reach the brain tumor site and exert anti-tumor effects, CAR-T cells treatment also needs to solve the problems of the tumor microenvironment and antigen heterogeneity to improve the efficacy of CAR-T cells in the treatment of glioblastoma. In 2019, Goff et al. reported another clinical trial of CAR-T cells targeting EGFRvIII in treating glioblastoma (NCT01454596) (72). In this clinical trial, no tumor regression was observed, 2 of the treated patients had severe hypoxia, and one had death. This clinical trial also reflects that the negative response associated with CAR-T cell therapy is a challenge that cannot be ignored.

In 2015, Sabrina Genßler et al. constructed a dual-specific CAR-NK cell targeting EGFR and EGFRvIII using NK92 as effector cells. The chimeric antigen receptor uses a CD28 and CD3ζ costimulatory domain. In the glioblastoma xenograft model, mice treated with dual-target-specific CAR-NK cells can effectively inhibit tumor growth and prolong the survival time of mice (24). In 2016, Nadja Müller et al. used YTS as an effector cell to overexpress chemokine receptor CXCR4 in CAR-NK cells targeting EGFRvIII, and the chimeric antigen receptor used DAP12 costimulatory domain. In the glioblastoma xenograft model, mice treated with CAR-NK cells also inhibited tumor growth and prolonged survival of mice (26). However, in 2020, Tsutomu Nakazawa et al. used KHYG-1 as an effector cell to construct CAR-NK cells targeting EGFRvIII, and chimeric antigen receptors used CD28,4-1BB and CD3ζ costimulatory domains. *In vitro* experiments, the cells can effectively kill EGFRvIII tumor cells. Still, in the animal model of immunodeficiency mice, the cell therapy cannot inhibit tumor progression and even induces pseudoprogressive pathological features (27). Although clinical trials of CAR-NK cells targeting EGFRvIII are still underway, the above studies have shown that different sources of NK cells and different molecular structures of chimeric antigen receptors can affect the therapeutic effect of CAR-NK cells.

### 3.3 IL-13Rα2

IL-13Rα2 is expressed on the cell membrane and is a subunit of the IL-13 receptor complex. Under physiological conditions, IL-13 is recruited and recognized by IL-13Rα1, and then IL-13Rα1 binds to IL-4Rα to form a heterodimer, which recruits Jak kinase and activates STAT6, thereby activating intracellular downstream signals (73) (74). Although IL-13Rα1 and IL-13Rα2 have the same binding mode for IL-13, the extracellular recognition domain of IL-13Rα2 has a unique receptor residue, which makes the IL-13 binding pocket of IL-13Rα2 larger, and the spatial complementarity between IL-13Rα2 and IL-13 is higher. Therefore, compared with IL-13Rα1, IL-13Rα2 has a higher affinity for IL-13 (75) (76). However, IL-13Rα2 lacks an intracellular domain and does not have a signal transduction role after recognizing IL-13 (77) (78). Studies have shown that IL-13Rα2 is highly expressed in glioblastoma, but its expression is low in normal brain tissue and other normal tissues. In contrast, patients with high expression of IL-13Rα2 have worse survival prognoses (79) (80) (81). Functionally, IL-13Rα2 competitively binds to IL-13, thereby blocking IL-13Rα1-mediated STAT6 phosphorylation, allowing tumor cells to escape apoptosis, so IL-13Rα2 is also called a pseudoreceptor (82). In addition, high expression of IL-13Rα2 also enhances the migration ability of tumor cells (83). Early clinical studies have shown that targeting IL-13Rα2 in the treatment of glioblastoma safety requirements and is a potential therapeutic target for glioblastoma (84) (85).

In 2015, Christine E. Brown et al. first reported the clinical experimental study of CAR-T targeting IL-13Rα2 in treating glioblastoma (NCT00730613) (86). In this clinical trial, 3 patients with recurrent glioblastoma were enrolled, and 12 intracranial orthotopic administrations were performed with a maximum dose of 10^8^ CAR-T cells. After CAR-T cells treatment, 2 of the 3 patients had a transient anti-tumor response. The expression of IL-13Rα2 in one patient was lower than before treatment, and the volume of tumor necrosis in the other patient was significantly larger after treatment. However, in this clinical study, all patients developed disease recurrence and eventually died after CAR-T treatment, and IL-13Rα2 antigen loss may be a potential mechanism leading to CAR-T cells treatment tolerance. In 2016, Christine E. Brown et al. reported another clinical experimental study of CAR-T cells targeting IL-13Rα2 in treating glioblastoma (NCT02208362) (87). In this study, only one patient with recurrent glioblastoma was enrolled and treated with multiple intracranial *in situ* administrations of CAR-T cells. After treatment, all intracranial and spinal tumors regressed, and no grade 3 or more toxic and side effects occurred during the period. The clinical treatment response lasted for 7.5 months. The above research laid the foundation for CAR-T cells targeting IL-13Rα2 in treating glioblastoma. Compared with CAR-T cells, there is still a lack of CAR-NK cells research reports targeting IL-13Rα2. In fact, in the absence of IFNγ, human NK cells secrete IL-13 cytokines under the stimulation of IL-2 (88) (89). Under the stimulation of IL-12, IL-13 can increase the expression of IFNγ in NK cells (90). In summary, we may suggest that CAR-NK cells targeting IL-13Rα2 can be used in treating glioblastoma in the future, and the problem of tumor escape caused by IL-13Rα2 antigen loss should be solved.

### 3.4 CSPG4

CSPG4, also known as glial antigen 2 (NG2), is a type I transmembrane protein expressed on the cell membrane and belongs to the chondroitin sulfate proteoglycan family. In tumor tissues, CSPG4 was first found to be highly expressed in melanoma tissues (91). Its expression is closely related to tumor cell proliferation and metastasis (92) (93) (94). CSPG4 is almost not expressed or low in normal tissues, but highly expressed in various solid tumor tissues (95) (96). CSPG4 is a marker of oligodendrocyte progenitor cells. In the state of disease, the expression of CSPG4 may be closely related to the occurrence and development of glioma. CSPG4 can induce cell proliferation, migration and tumor angiogenesis. The expression of CSPG4 can be detected in oligodendroglioma, astrocytoma and glioblastoma (97). Recent studies have shown that CSPG4 is highly expressed in about 50% of glioblastomas, and the expression of CSPG4 can be used as an independent factor for the prognosis of patients. Patients with high expression of CSPG4 have a poorer survival prognosis (98). Compared with tumor-associated antigens such as Her2 and IL-13Rα2, CSPG4 is expressed higher in glioblastoma cells, and CSPG4 is also found to be highly expressed in tumor-associated perivascular cells (99). Although no relevant clinical trials have been carried out, preclinical studies have shown that tumor growth was significantly inhibited in mice treated with CSPG4-specific antibodies (95) (94).

In 2018, Serena Pellegatta et al. constructed CAR-T cells targeting CSPG4. In the patient-derived glioblastoma model constructed by immunodeficient mice. After intratumoral administration of the CAR-T cells, the tumor progression with high expression of CSPG4 and moderate expression of CSPG4 was inhibited, After treatment, the tumor burden of mice decreased, the survival time was significantly prolonged, and about 60% of mice survived in long-term observation (99). Further studies have shown that TNFα secreted by CAR-T cells induces CSPG4 expression in glioblastoma, thereby avoiding tumor escape due to antigen loss (99). The glioblastoma model was constructed in immunodeficient mice in the study based on NK cells. After the combination of NK cells and anti-CSPG4 monoclonal antibody mAb9.2.27, the glioblastoma tumor progression was inhibited and survival time of the mice was significantly prolonged (100). Subsequent studies have shown that activated NK cells secrete IFNγ, activating and recruiting macrophages/microglia to tumor tissues. Activated macrophages/microglia have a killing effect on glioblastoma tumor cells, and their killing ability is even better than NK cells. Moreover, the inhibition of tumors by NK cells and mAb9.2.27 combined treatment depends on the participation of macrophages/microglia (100). The above studies have shown that NK cells targeting CSPG4 are feasible for treating glioblastoma, and there is no relevant report. At the same time, tumor infiltration of macrophages/microglia is also a key factor affecting treatment response, and related combination therapy may be a more effective treatment strategy.

### 3.5 GD2

Ganglioside is a complex glycolipid on the cell membrane. It mainly exists in the nervous system and is a natural component of the neuronal cell membrane (101) (102). GD2, namely disialoganglioside GD2. In cells, GD2 is synthesized in the endoplasmic reticulum and Golgi apparatus and then transferred to the cell membrane. Functionally, GD2 is closely related to cell adhesion and signal transduction. GD2 plays a crucial role in physiological and pathological processes by driving cell proliferation, angiogenesis and immune escape. The primary pathological significance of GD2 is that it is highly expressed in various malignant tumors (103) (104) (105). In a study of brain tumors, the positive expression of GD2 was found in 80% of diffuse pontine gliomas (106). In addition, glioblastoma tumor stem cells also found positive expression of GD2 (107). In 2021, Malvina Prapa et al. successfully isolated tumor cells from 12 patients with glioblastoma, of which 7 patients had high expression of GD2, and the positive rate of GD2 was more than 80% (108). At the same time, the study also constructed GD2-specific CAR-T cells, and the results showed that GD2-specific CAR-T cells could exert anti-glioma activity *in vivo* and *in vitro*. This study suggests that GD2 is a potential target for CAR-T cells in treating glioblastoma.

In 2022, Robbie G. Majzner et al. first reported a clinical trial of GD2-specific CAR-T cells in treating glioma (NCT04196413) (109). H3K27M mutant glioma cells highly express GD2. Four patients with H3K27M mutant glioma were enrolled in this clinical trial and treated with intravenous infusion of GD2-specific CAR-T cells. Subsequently, patients who benefited from the treatment continued to infusion GD2-specific CAR-T cells intraventricular. In this clinical trial, 3 of the 4 patients treated with GD2-specific CAR-T showed clinical and radiographic improvement. The results of this study indicate that GD2-specific CAR-T cells have certain clinical benefits in the treatment of glioma. Although there is no report on the treatment of glioblastoma with GD2-specific CAR-NK cells, GD2-specific CAR-NK cells have shown specific anti-tumor effects on neuroblastoma in preclinical studies (110). The above studies suggest that GD2-specific CAR-NK cell is a potential therapeutic strategy for GD2-positive glioblastoma, and related research can be carried out in the future.

## 4 Challenges of CAR-NK cell therapy for glioblastoma

As mentioned above, CAR-NK cells are feasible for the treatment of glioblastoma. Compared with CAR-T cells, CAR-NK cells have the advantages of safety. Similar to CAR-T cells, CAR-NK cells also need to solve the problems of tumor heterogeneity and tumor immune microenvironment inhibition in treating solid tumors such as glioblastoma. In addition, CAR-NK cells also have challenges such as low preparation efficiency, short survival time *in vivo*, and optimization of anti-tumor activity.

### 4.1 The immune microenvironment of glioblastoma

**Figure 1:**
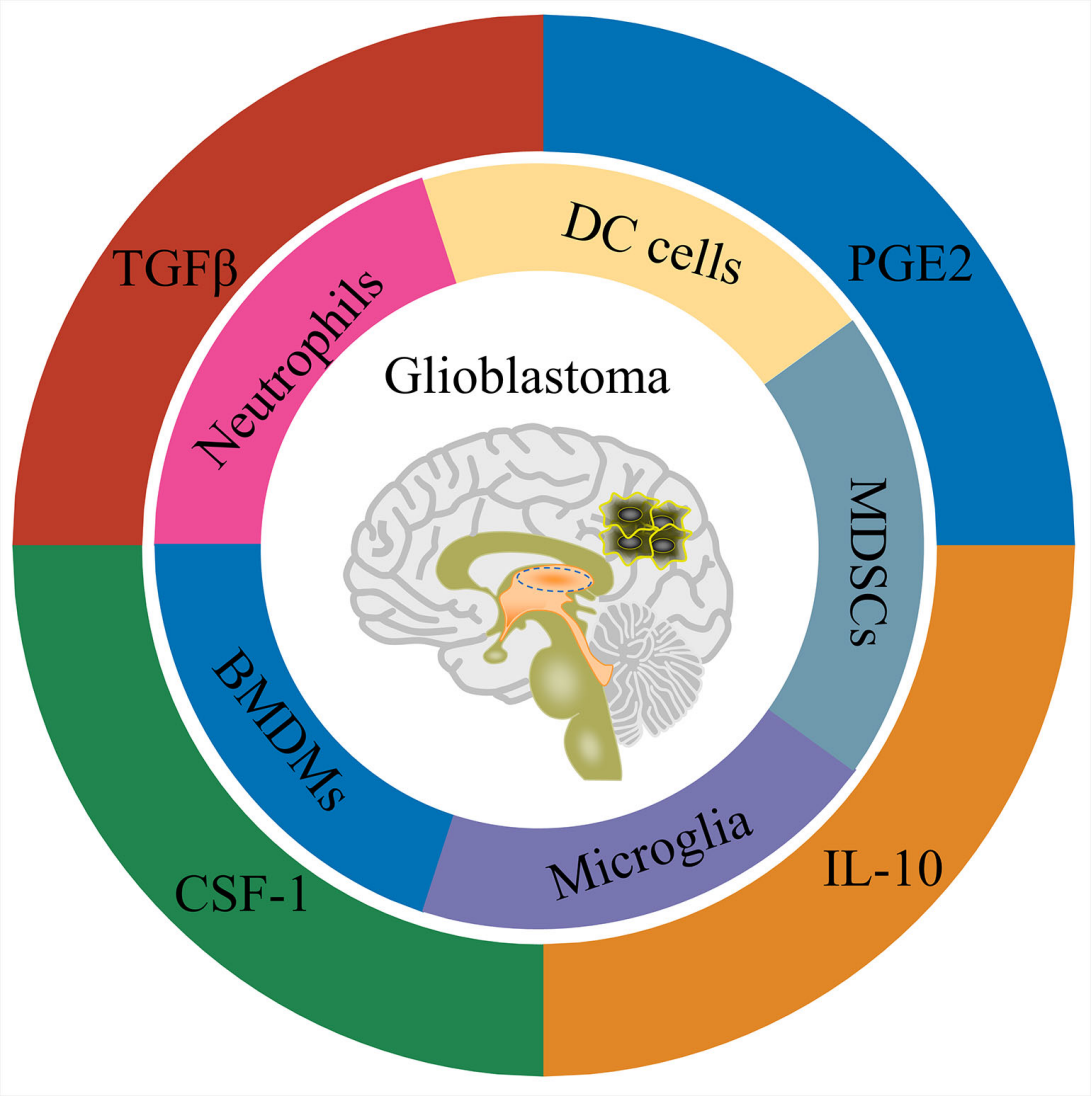
Schematic representation of GBM tumor microenvironment.

As for the blood-brain barrier, the brain has long been considered immune-protected tissue (111). Along with the researches, the brain is now more inclined to be regarded as an immune-specific tissue (112). There are many different types of immune cells in the central nervous system, and they also have functional lymphatic vessels. Glioblastoma is a ‘ cold tumor ‘ due to the lack of lymphocyte infiltration (113). There are mainly four types of cells in the immune microenvironment of glioblastoma, namely: tissue resident cells, such as neurons and astrocytes; myeloid-derived immune cells (**Figure 1**), such as tissue-resident microglia, bone marrow-derived macrophages (BMDMs), bone marrow-derived DC cells, bone marrow-derived suppressor cells (MDSCs) and neutrophils; the lymphoid-derived immune cells, such as T cells and NK cells; other cells, such as endothelial cells, pericytes, and fibroblasts (114) (115). Weilun Fu et al. used single cell technology to systematically analyze the immune cells in the glioblastoma tumor microenvironment (13). The study found that in the tumor microenvironment of glioblastoma, myeloid-derived immune cells accounted for a relatively high proportion, most of which were tumor-associated macrophages/microglia. In addition, there is a small amount of infiltration of T cells and NK cells in the glioblastoma tumor microenvironment. Still, the T cells are mainly Treg cells, as well as exhausted phenotype helper T cells and cytotoxic T cells, while the NK cells are non-functional NK cells.

Consistent with the cell type, there are many immunomodulatory molecules in the tumor microenvironment of glioblastoma, including TGFβ, IL-10, IL-6 and PGE2 (**Figure 1**). In addition, cells in the glioblastoma tumor microenvironment up-regulate the expression of immune checkpoint receptor-related molecules, such as PD-1/PD-L1, CTLA-4/CD80/CD86 and TIM-3/galectin-9 (115). In summary, glioblastoma is an immunosuppressive ‘cold tumor’, posing great challenges for tumor immunotherapy such as CAR-T and CAR-NK cells. Fortunately, tumor-associated macrophages/microglia are also involved in the anti-tumor effect of NK cell-based immunotherapy (100). In a study of chimeric antigen receptor CAR-NK cells based on NKG2D design, the CAR-NK cells could tolerate the inhibitory effect of TGFβ or soluble MICA/MICB on cytotoxic function in the immune microenvironment. In contrast, the cytotoxicity of wild-type NK cells was inhibited under the condition of TGFβ or soluble MICA/MICB (116). Recent studies have shown that TGFβ signaling is crucial for glioblastoma tumor stem cells escaping NK cell immune surveillance (117). Inhibition of TGFβ signaling or knockout of TGFβ receptor TGFBR2 can prevent NK cell dysfunction and enhance the anti-tumor effect of NK cells on glioblastoma stem cells (117). This study suggests that blocking TGFβ signaling may also improve the anti-tumor effect of CAR-NK cells on glioblastoma. By co-transducing a mutant TGFBR2 (DNR) for B7H3-specific CAR-NK cells, Kajal Chaudhry et al. showed that this co-transducing CAR-NK cells could tolerate the immunosuppressive effect of exogenous TGFβ and further enhance the anti-tumor effect of CAR-NK cells on glioblastoma (118). The above studies suggest that blocking TGFβ signaling is an alternative strategy for CAR-NK cells to glioblastoma therapy. Nevertheless, only a few CAR-NK cell studies have considered the effect of immunosuppressive microenvironment on CAR-NK function. From the perspective of clinical application, more research should be conducted on how CAR-NK cells overcome the immunosuppressive microenvironment of solid tumors such as glioblastoma in the future.

### 4.2 The preparation of CAR-NK cells

The preparation of CAR-NK cells includes two parts: the culture of NK cells and the genetic modification of NK cells. These two parts are also the main challenges in CAR-NK cell preparation. At present, the sources of NK cells used to prepare CAR-NK are abundant, mainly from four types of NK cells, namely NK-derived tumor cell line (NK92 et al.), peripheral blood-derived NK cells (PB-NK), umbilical cord blood-derived NK cells (CB-NK) and iPSC-derived NK cells (iPSC-NK). NK cells from different sources have their advantages and disadvantages (**Table 2**) (14) (35). For example, NK92 cells can be cultured on a large scale and have low immunogenicity, but NK92 is a tumor-derived cell line with a risk of tumorigenicity in clinical applications. PB-NK has a wide range of sources and high safety, but the efficiency of PB-NK culture *in vitro* is low. At present, iPSC-NK is also a research hotspot in the field and has made some breakthroughs (119) (120). Moreover, CAR-NK cells prepared from different sources of NK cells have other functions. Stephan Kloess et al. showed that CAR-NK92 has more potent anti-tumor cytotoxicity than CAR-PB-NK, but CAR-NK92 also has more potent cytotoxicity to non-tumor cells, suggesting that CAR-NK92 may cause side effects of ‘on-target’ non-tumor cells (39). Herrera et al. showed that CAR-PB-NK had stronger anti-tumor cytotoxicity than CAR-CB-NK, but CAR-CB-NK had stronger proliferation activity (121). In addition to the above-mentioned sources of NK cells, Han-Seop Kim et al. have recently developed a direct somatic reprogramming source of NK cells (drNK). Compared with PB-NK, ES-NK and iPSC-NK cells, drNK is a specific phenotype of CD56^bright^CD16^bright^NK cells. Compared with CAR-PB-NK, CAR-drNK has a stronger killing effect on tumor cells and tumor stem cells (122). In the preparation cycle, a large amount of drNK can be obtained in 24 days from the initial culture. Therefore, drNK is another potential source of NK cells.

**Table 2:** Comparison of NK cells from different sources.

NK Cells	Advantages	Disadvantages
NK92	1. Easy to culture *in vitro*. 2. Low expression of inhibitory receptors KIRs. 3. Low immunogenicity.	1. Lack of activating receptors. 2. Tumor-derived cells. 3. Risk of EBV infection.
PB-NK	1. Mature phenotype. 2. High safety.	1. Low amplification efficiency *in vitro*. 2. Short survival time *in vivo*.
CB-NK	1. Rich source. 2. High safety.	1. Low amplification efficiency *in vitro*. 2. Short survival time *in vivo*. 3. High expression of inhibitory receptor NKG2A. 4. Lack of ADCC function.
iPSC-NK	1. Rich source. 2. Cloning screening can be carried out. 3. Facilitate genetic modification. 4. Low expression of inhibitory receptors KIRs.	1. High expression of inhibitory receptor NKG2A. 2. Short survival time *in vivo*.

In addition to NK92 cells, NK cells from other sources face the problem of low *in vitro* culture efficiency. At present, the culture of NK cells is divided into two technical routes, one is the feeder cell stimulation culture method, and the other is the non-feeder cell culture method (123) (124). The non-feeder cell culture method activates NK cells through different cytokine combinations and maintains the proliferation of NK cells. The feeder cell method activates NK cells through engineered tumor cells and maintains the proliferation of NK cells under the stimulation of cytokines. Compared with the non-feeder cell culture method, the feeder cell culture method has higher NK cell culture efficiency (124). In the technical route of feeder cell culture method, K562-4-1BBL-mbIL21 was used as feeder cells. After 21 days of culture by Cecele J. Denman et al., NK cells proliferated 31747 times (125), the highest amplification efficiency observed in the literature. Mechanistically, feedeer cell culture provides three necessary signals for NK cell proliferation, namely cell-cell contact, CD137 signal and cytokine signal (126). The feeder cell culture method solves the problem of NK cell culture *in vitro*, but most of the feeder cells used for NK cell culture are tumor-derived cell lines. The NK cells cultured by this method have potential safety risks when reinfusion *in vivo*. Currently, the methods of mitomycin, γ-ray irradiation and freeze-thaw are often used to inactivate feeder cells (127) (128), which alleviates the safety concerns to a certain extent. However, there is a lack of systematic research on the safety evaluation of NK cells by feeder cell culture. Therefore, safe and efficient NK cell culture is still challenging in NK cell therapy.

The genetic modification of NK cells is another challenge. At present, the genetic modification of NK cells mainly includes two methods: electroporation and viral transduction (14). Compared with viral transduction, electroporation is more efficient (129). Lin Xiao et al. used electroporation to transfect NK cells in the study of CAR-NK cells. The transfected nucleic acid was mRNA that overexpressed chimeric antigen receptors, and the transfection efficiency of NK cells in this study was about 100% (130). Although the efficiency of electroporation is high, it will reduce cell viability, and the genetic modification of electroporation is transient. Despite this, there have been many successful reports on electroporation technology in CAR-T cells preparation (131) (132) (133). Therefore, electroporation technology will also make a breakthrough in CAR-NK cells. Currently, viral transduction is still widely used in CAR-NK cell research, and viral transduction is also a recognized method in CAR-T cell applications. Since the widely used pseudovirus is VSV-G envelope protein lentivirus, the cell receptor of VSV-G is LDLR, and the expression of LDLR in NK cells is low, which also leads to the low transduction efficiency of VSV-G envelope protein lentivirus on NK cells (134). To solve this problem, researchers have tried to use viruses with different envelope proteins to modify NK cells, among which RD114 envelope protein retrovirus and BaEV envelope protein lentivirus are more successful (134) (135). The typical cell receptor of these two envelope proteins is ASCT2, which is highly expressed in activated NK cells (134). Compared with RD114, the cell receptor of BaEV contains another receptor ASCT1, so the efficiency of BaEV envelope protein lentivirus transduction of NK cells is higher (136). However, NK cells are a class of natural antiviral immune cells that have a natural resistance to viral transduction, which is one of the reasons for the low efficiency of viral transduction of NK cells. Tolga Sutlu et al. used the BX795 inhibitor to block TBK1/IKKϵ, which can partially inhibit the antiviral response of NK cells, thereby increasing the viral transduction efficiency by 3.8 times (137). Peter Chockley et al. obtained a similar conclusion using inhibitors such as BX795, that is, blocking TBK1/IKKϵ can improve the efficiency of virus transduction of NK cells (138). The genetic modification efficiency of NK cells was effectively improved by modifying the envelope protein and blocking TBK1/IKKϵ. However, future research still needs to address the problems of low virus packaging efficiency and cytotoxicity of inhibitors such as BX795.

### 4.3 The persistence of CAR-NK cells

The short survival time of NK cells *in vivo* is a significant challenge for adoptive NK cell therapy. In clinical studies of adoptive NK cell therapy, NK cells survive within 2 weeks *in vivo* (139) (140) (141). The short residence time of NK cells in the body can bring safety advantages, but it also limits the therapeutic efficiency of NK cells. At present, cytokines such as IL-2 and IL-15 are often used to prolong the survival time of NK cells *in vivo* (123). In the study of CAR-NK cells, to extend the survival time of CAR-NK cells *in vivo* and improve its therapeutic efficiency, the same strategy will be used, that is, IL-2 or IL-15 adjuvant therapy (20) (142) (143) (144) (145) (146). Although IL-2 is more widely used, IL-15 may be more effective in maintaining NK cell survival *in vivo*. Elizabeth L. Siegler et al. evaluated the survival of CAR-NK cells *in vivo* under cytokine support therapy. Animal experiments were designed to give IL-15 support therapy 1-7 days after CAR-NK cells treatment and IL-2 support therapy 1-21 days after CAR-NK cells treatment. The proportion of NK cells in peripheral blood, spleen and ascites was detected at different times after CAR-NK cells treatment. The results showed that the proportion of NK cells in various tissues reached the maximum on the day10 and then gradually decreased (20). This study indicates that IL-15 has a higher efficiency in maintaining the survival of NK cells *in vivo* than IL-2. Similarly, Enli Liu et al. constructed CAR-NK cells with endogenous expression of IL-15, and continuously detected the peripheral blood of CAR-NK cells treated mice. It was found that CAR-NK cells continued to survive in the peripheral blood at a high proportion until day 49, and a certain proportion of CAR-NK cells could still be detected on the 75th day after treatment (146). This study further confirmed that IL-15 can effectively maintain the survival of NK cells *in vivo*.

Although cytokines can prolong the survival time of NK cells *in vivo*, cytokine support therapy may also bring corresponding side effects. It has been confirmed that systemic IL-2 treatment can induce the activation of Treg cells and bring serious vascular leakage syndrome and neurotoxicity and other side effects (147) (148) (149). Systemic administration of IL-15 mainly affects NK cells, γδ cells and CD8 memory T cells, but IL-15 can cause symptoms such as hypotension and thrombocytopenia in a dose-dependent manner and can lead to a decrease in neutrophils (150). Compared with IL-2, IL-15 is a safer choice (151) (152). However, the systemic toxicity of IL-15 cannot be ignored. Recent studies have shown that after CAR-NK cells co-express IL-15, mice treated with the CAR-NK cells can cause systemic toxicity in animal models of acute lymphoblastic leukemia. Although CAR-NK cells have the most potent anti-tumor effect *in vivo*, the survival of mice has not been significantly improved (153). In summary, IL-15 has a significant advantage in maintaining the survival of NK cells *in vivo*, but the systemic toxicity caused by IL-15 cannot be ignored. To make better use of this characteristic of IL-15, the safe dosage and administration mode of IL-15 can be further studied in the future. In addition to natural cytokines, directional modified IL-2 cytokines have a high affinity for IL-2Rβ receptors and can specifically activate T cells (154) (155). Studies have shown that EPO/TPO can improve the survival of NK cells *in vivo* and enhance their anti-tumor activity (156). At present, there are few reports on the continued survival of CAR-NK cells. We may suggest that the future can focus on this progress.

### 4.4 The anti-tumor activity of CAR-NK cells

**Figure 2:**
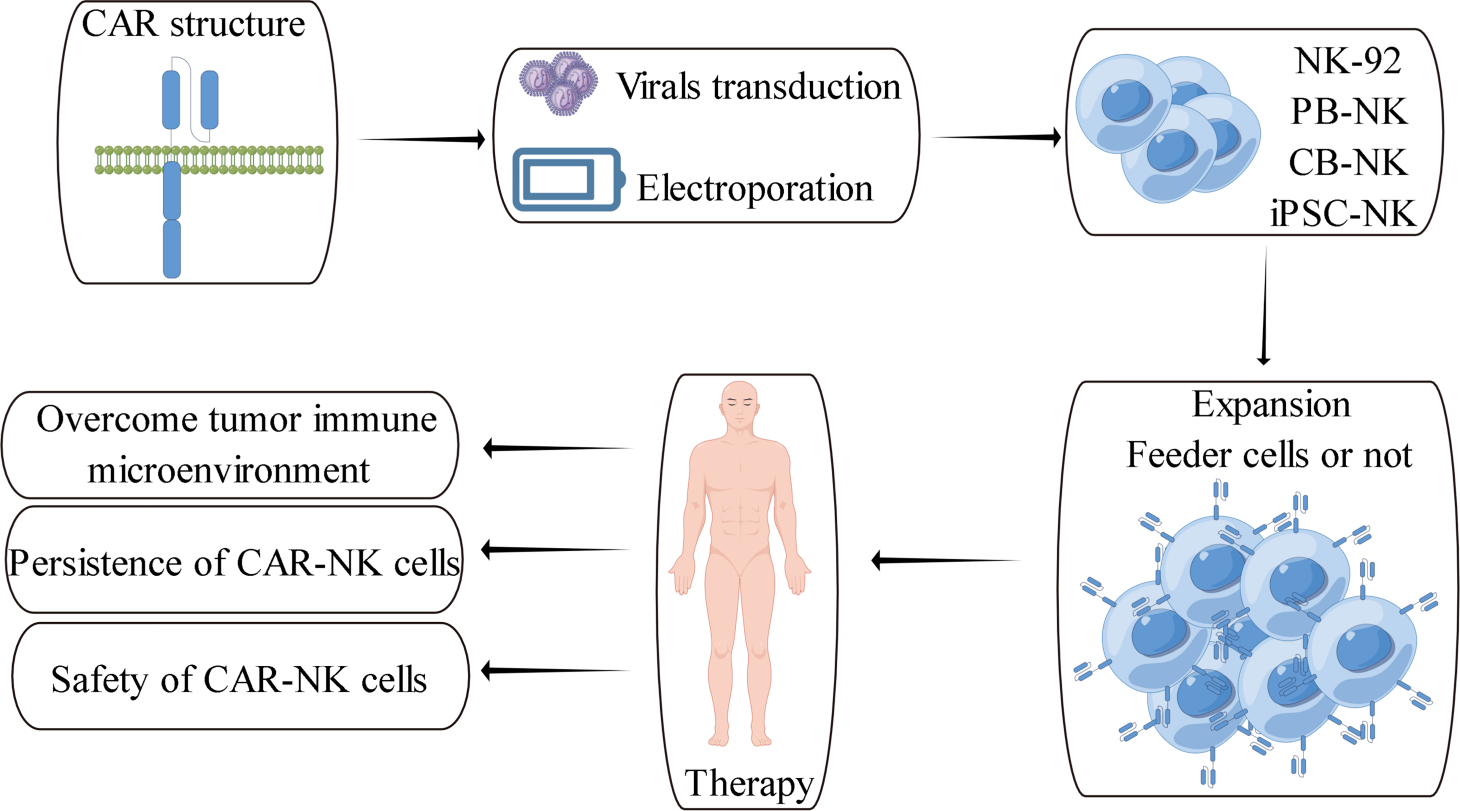
Schematic overview of CAR-NK cell therapy and its challenges (157).

The classical chimeric antigen receptor contains four components: extracellular antigen recognition domain, hinge region, transmembrane domain and intracellular domain (**Figure 2**). The antigen recognition domain is usually composed of single chain antibody (scFv). The hinge region is used to connect the antigen recognition domain and the transmembrane domain. Its length plays an vital role in the formation of immune synapses by effector cells. Currently, the hinge region of CAR-NK cells primarily uses the hinge region fragment of CD8α, while the transmembrane domain of CAR-NK cells mainly uses the transmembrane domain fragment of CD28. Compared with the above components, the intracellular costimulatory domain of chimeric antigen receptors is essential for the activation and function of effector cells. Currently, the main costimulatory domain used in CAR-NK cell research is CD28-CD3ζ (14) (157). CD28-CD3ζ is one of the intracellular costimulatory domains often used in classical second-generation CAR-T cells. With the deepening of CAR-NK cells research, more and more evidence shows that it is not the best choice to construct CAR-NK cells directly using CAR-T-derived chimeric antigen receptors (14). The activation of NK cells and T cells are quite different. The activation of T cells mainly depends on their specific TCR receptors, while the activation of NK cells depends on various activating receptors expressed on their cell membranes (18). Therefore, it is necessary to rationally design chimeric antigen receptors based on the characteristics of NK cells, and CAR-NK cells based on rational design have also made positive research progress.

Through a comprehensive analysis of NK cell activating receptors, Ece Canan Sayitoglu et al. found that NK cells overexpressing DNAM1 or NKG2D can enhance the killing effect of NK cells on tumor cells (158). DNAM1 is an activating receptor expressed on the cell membrane of NK cells, which activates NK cells after recognizing ligands CD155 and CD112 (159) (160). DNAM1 is a type I transmembrane protein. Ming-Ru Wu et al. linked DNAM1 to the intracellular costimulatory domain of CD3ζ to form a chimeric receptor. By comparison, NK cells expressing chimeric receptors have a better killing effect on tumor cells than NK cells expressing wild-type DNAM1 receptors. However, NK cells expressing chimeric receptors will reduce their anti-tumor effects after adding a costimulatory domain CD28, OX40 or 4-1BB between DNAM1 and CD3ζ (161). In 2020, Yao Huang et al.added 2B4 costimulatory domain between DNAM1 and CD3ζ. Compared with CD3ζ, CD28-CD3ζ, DNAM1-CD3ζ and 2B4-CD3ζ, CAR-NK expressing DNAM1-2B4-CD3ζ chimeric antigen receptor has the best anti-tumor effect (162). The above studies have shown that the expression of DNAM1 can enhance the anti-tumor effect of NK cells. In the construction of chimeric antigen receptors, DNAM1-2B4-CD3ζ is an ideal choice, and further research can be carried out on this basis.

In addition to DNAM1, Ece Canan Sayitoglu et al. also confirmed that NK cells overexpressing NKG2D can enhance the killing effect of NK cells on tumor cells (158). NKG2D is an activating receptor expressed on the cell membrane of NK cells, and its ligands are MICA, MICB and ULBPs (163) (164). Unlike type I transmembrane proteins such as CD28, 4-1BB, DNAM1 and CD3ζ, NKG2D belongs to type II transmembrane protein. Compared with type I transmembrane protein, the transmembrane expression of type II transmembrane protein does not require the guidance of signal peptide, and the C-terminal of type II transmembrane protein is outside the cell membrane. Therefore, two different strategies exist for modifying chimeric receptors based on NKG2D. First, without changing the characteristics of NKG2D type II transmembrane protein, the added costimulatory domain is connected to the N-terminus of NKG2D, or the extracellular recognition domain is linked to the C-terminus of NKG2D. Robin Parihar et al. linked an intracellular costimulatory domain of CD3ζ to the N-terminus of NKG2D. Unlike unmodified NK cells, NK cells modified with this chimeric receptor have more potent anti-tumor effects. Moreover, NK cells modified with this chimeric receptor can tolerate the interference of TGFβ and soluble MICA/MICB on the anti-tumor effect of NK cells in the immune microenvironment (116). Using a similar strategy, Changjiang Guo et al. connected a 4-1BB costimulatory domain to the N-terminus of NKG2D. Compared with the chimeric receptor without the 4-1BB costimulatory domain, NK cells modified with this chimeric receptor have more potent anti-tumor effects. This study also confirmed that modifying the C-terminal extracellular recognition domain of NKG2D would directly determine the function of chimeric receptors when using this strategy to modify NKG2D (165).

Another modification strategy for NKG2D is to use only the transmembrane domain of NKG2D, and design it from scratch according to type I transmembrane protein design method. The signal peptide, recognition domain and hinge region are linked at the N-terminus of the transmembrane domain of NKG2D, and different costimulatory domains are linked at the C-terminus of the transmembrane domain of NKG2D. NKG2D induces cell activation by forming a heterohexamer with DAP10, and the binding of NKG2D to DAP10 depends on the arginine on the NKG2D transmembrane domain (166) (167). Therefore, this strategy effectively retains the functional domain of NKG2D, namely the transmembrane domain. More representative is a 2018 study by Ye Li et al. (20). The study used this strategy to add different combinations of costimulatory domains at the C-terminus of the NKG2D transmembrane domain. The chimeric antigen receptor with costimulatory domain 2B4 and CD3ζ have the best anti-tumor effect after modifying NK cells, and its anti-tumor effect is better than that of NK cells expressing T-CAR binding antigen receptors. In the tumor xenograft model of immunodeficient mice, compared with CAR-T cell therapy, mice treated with CAR-NK cells had longer survival. It is worth noting that when the different domains of the chimeric antigen receptor were functionally verified, the inactivation mutation of CD3ζ did not significantly reduce the killing effect of CAR-NK on tumor cells. In another study, Lin et al. compared DAP12 with CD3ζ and found that NK cells using DAP12 costimulatory domain chimeric antigen receptor had more potent anti-tumor effect than those using CD3ζ costimulatory domain chimeric antigen receptor (130). This study suggests that DAP12 is more suitable for designing chimeric antigen receptors than CD3ζ in constructing NK cell chimeric antigen receptors. The above studies have shown that NKG2D is a vital receptor to enhance the anti-tumor activity of NK cells no matter which strategy is used. When modifying NKG2D, the properties of its type II transmembrane protein should be fully considered for reasonable design and strict verification.

It is worth noting that both DNAM1 and NKG2D can effectively enhance their anti-tumor activity when combined with 2B4 in the construction of chimeric antigen receptors. When 2B4 is not combined with DNAM1 or NKG2D, the design of chimeric antigen receptors based on 2B4 can also improve the anti-tumor activity of NK cells. In 2019, Yingxi Xu et al. compared 2B4-CD3ζ with 4-1BB-CD3ζ. This study showed that NK cells using 2B4 costimulatory domain chimeric antigen receptors had more potent anti-tumor effects (168). In 2021, Ilias Christodoulou et al. also confirmed the same conclusion, that is, compared with 4-1BB-CD3ζ, NK cells using 2B4 costimulatory domain chimeric antigen receptor have more potent anti-tumor effect (153). Therefore, 2B4 is also a vital candidate receptor when designing NK cell chimeric antigen receptors.

In summary, the rational design of NK cell chimeric antigen receptors is of great significance for improving the anti-tumor activity of CAR-NK cells. DNAM1, NKG2D and 2B4 are essential candidates for rational design. In addition, the mechanism of the combined action between different domains of chimeric antigen receptors also needs to be further elucidated.

### 4.5 The safety of CAR-NK cells

Compared with CAR-T cell therapy, CAR-NK cell therapy is more safe (14). In 2018, Ye Li et al. used iPSC-derived NK cells as effector cells to construct mesothelin-targeted CAR-NK cells for treating human ovarian cancer (20). In this preclinical study, the survival time of mice treated with CAR-NK cells was significantly prolonged, and no apparent cytokine storm was detected during the treatment. As a control, CAR-T cell therapy slightly improved the survival of mice. The survival rate of mice in the CAR-T cells treatment group was significantly lower than that in the CAR-NK cells treatment group. In the same year, Enli Liu et al. used umbilical cord blood-derived NK cells as effector cells to construct CAR-NK cells targeting CD19 for treating lymphoblastic leukemia (146). In this preclinical study, the survival time of mice treated with CAR-NK cells was significantly prolonged. After 10 months of treatment, pathological examination of lymph nodes, spleen and bone marrow of mice showed no abnormal proliferation of CAR-NK cells. Subsequently, in 2020, Enli Liu et al. reported the clinical results of umbilical cord blood CAR-NK cells in treating lymphoblastic leukemia (22). A total of 11 patients were enrolled in this clinical trial. After CAR-NK cell administration, 8 patients showed a therapeutic response, of which 7 patients were completely relieved. After CAR-NK cells treatment, there was no treatment-related cytokine storm, neurotoxicity and anti-host disease, and the levels of inflammatory factors such as IL-6 did not exceed the baseline level. The above studies have shown that CAR-NK treatment has safety advantages.

The concerns about the safety of CAR-NK cells come from two aspects. First, NK-derived tumor cell lines are used as effector cells in many preclinical and even some clinical studies (14), and there is a risk of tumorigenicity. The current solution is to use gamma-ray irradiation and other methods to treat NK-derived tumor cell lines to maintain cell activity and inhibit their proliferation. In 2017, Paulina Nowakowska et al. constructed the preparation of clinical-grade CAR-NK92 cells. This study used NK-derived tumor cell line NK92 cell as effector cells. The prepared CAR-NK92 cells were irradiated with 10 Gy gamma-ray irradiation. This study showed that CAR-NK92 cells lost the ability of cell proliferation after irradiation, and CAR-NK92 cells could maintain their cell activity and function within 24 h after treatment (36). In 2020, Qian Liu et al. further studied the irradiation dose. In this study, CAR-NK92-MI cells were also irradiated with different doses of gamma rays. Compared with 10 Gy irradiation dose, CAR-NK92-MI cells irradiated with 5 Gy irradiation dose showed better anti-tumor ability in mice, and the survival of mice was improved more significantly (38). Compared with gamma-ray irradiation, treating CAR-NK92 cells with low-energy electron irradiation can also effectively inhibit the proliferation of CAR-NK92 cells, and has less effect on the whole gene expression level of cells (37).

Although methods such as gamma-ray irradiation can effectively inhibit the proliferation activity of NK-derived tumor cell lines, different NK-derived tumor cell lines have different functions in anti-tumor effects. Tsutomu Nakazawa et al. used NK-derived tumor cell line KHYG-1 as an effector cell to construct CAR-NK cells. In the glioblastoma animal model, the tumor progression of mice treated with CAR-NK cells was not inhibited, and even pseudoprogression occurred. In addition, the CAR-NK cells were co-cultured with tumor cells *in vitro*. High levels of IL-6 expression were detected, and IL-6 is one of the markers of cytokine storms (27). Therefore, in constructing CAR-NK cells using NK-derived tumor cell lines, in addition to inactivating cells such as irradiation to inhibit their tumorigenicity, the differences in anti-tumor effects between different NK-derived tumor cell lines and the resulting safety issues should be considered.

In some studies of CAR-NK cells, cytokines such as IL-2 are used to prolong the survival of CAR-NK cells *in vivo* and enhance the function of CAR-NK cells. Therefore, another concern about the safety of CAR-NK cells is the systemic toxicity these cytokines may bring to the body. Compared with IL-2, IL-15 is a safer choice (151) (152). Enli Liu et al.co-expressed IL-15 in CAR-NK cells to improve the anti-tumor effect of CAR-NK and prolong the residence time of CAR-NK cells *in vivo* (146). However, recent studies have shown that in animal models of CAR-NK cells co-expressing IL-15 in treating acute lymphoblastic leukemia, mice treated with CAR-NK cells can cause systemic toxicity. Although the CAR-NK cells have the most potent anti-tumor effect *in vivo*, the survival time of mice has not been significantly improved (153). In fact, in order to enhance the safety of CAR-NK cells, Enli Liu et al. introduced a suicide gene as a safety switch while co-expressing IL-15 (146). Therefore, the relatively safe IL-15 also needs to consider its potential safety issues. We may suggest that future research should rationally design CAR-NK cells treatment programs to enhance the anti-tumor effect of NK cells while overcoming the systemic toxicity of these cytokines.

## 5 Summary and prospect

Glioblastoma is a malignant tumor with the highest morbidity and mortality in the central nervous system. The survival time of patients is short, and the 5-year survival rate is less than 10%. Immunotherapy represents a promising class of glioblastoma treatment methods, such as tumor vaccines, immune checkpoint inhibitors, combination therapy and adoptive immunotherapy. Immunotherapy has carried out clinical research on glioblastoma (169). In adoptive cell immunotherapy, CAR-NK cell is another potential adoptive cell therapy strategy after CAR-T cell. Compared with CAR-T cells, CAR-NK cells do not cause similar adverse effects of CAR-T cell, such as cytokine storm and anti-host immune disease and has higher safety. In addition, the constructed CAR-NK cells retained the natural anti-tumor response of NK cells while allowing NK cells to specifically target tumors. CAR-NK cells with specific optimization have anti-tumor effects equivalent to CAR-T cells (20). At present, CAR-NK cell has achieved some positive therapeutic effects in preclinical studies of glioblastoma (**Table 1**), and clinical studies of CAR-NK cells targeting Her2 in the treatment of glioblastoma are also underway (NCT03383978).

However, CAR-NK cell treatment of glioblastoma also needs to address the inhibition in the immunosuppressive microenvironment of glioblastoma. For example, TGFβ in the immunosuppressive microenvironment is a classic immunomodulatory molecule. TGFβ plays a tumor-promoting role by inhibiting the anti-tumor activity of T cells and NK cells (170). In addition, CAR-NK cell faces challenges in terms of low preparation efficiency, short survival time *in vivo*, optimization of anti-tumor activity and safety. Fortunately, there are some solutions to these challenges of CAR-NK cell, such as the efficient preparation of CAR-NK cells *in vitro* by feeder-cells method or cytokine-assisted therapy to prolong the survival time of CAR-NK cells *in vivo*. Although these solutions are imperfect, they also lay the foundation for further research on CAR-NK cell therapy. In conclusion, CAR-NK cell is feasible in treating glioblastoma and a safe and effective cell therapy strategy. Future research should focus on optimizing the anti-tumor effect of CAR-NK and overcoming the tumor immunosuppressive microenvironment of solid tumors such as glioblastoma. CAR-NK has begun to see the dawn, and the future can be expected.

## Author contributions

QX drafted and proofread the manuscript. HD and JZ proofread the manuscript. YZ drew the picture of the manuscript. All authors contributed to the article and approved the submitted version.
